# The impact of empagliflozin on cardiac physiology and fibrosis early after myocardial infarction in non-diabetic rats

**DOI:** 10.1186/s12933-021-01322-6

**Published:** 2021-07-02

**Authors:** Elias Daud, Offir Ertracht, Nadav Bandel, Gassan Moady, Monah Shehadeh, Tali Reuveni, Shaul Atar

**Affiliations:** 1 The Cardiology Department, Galilee Medical Center, P.O. Box 21, 2210001 Nahariya, Israel; 2 Eliachar Research Laboratory, Galilee Medical Center, Nahariya, Israel; 3 Azrieli Faculty of Medicine, Bar-Ilan University, Safed, Israel

**Keywords:** Collagen deposition, Empagliflozin, Fibrosis, Myocardial infarction, TGF-β1**/**Smad3 pathway

## Abstract

**Background:**

Myocardial fibrosis is a multistep process, which results in collagen deposition in the injured muscle. Empagliflozin, a sodium-glucose cotransporter 2 inhibitor (SGLT2i), decreases cardiovascular events risk. Little is known on the effects of empagliflozin in non-diabetic patients early post myocardial infarction.

**Methods:**

Fourteen non-diabetic rats underwent myocardial infarction induction, and treated or not (control)immediately after myocardial infarction by daily empagliflozin (30 mg/kg/day). We evaluated cardiac function at baseline, 2 and 4 weeks after myocardial infarction by echocardiography, and prior to sacrifice by Millar pressure–volume system. We performed histological and biochemical evaluation of fibrosis and humoral factors promoting fibrosis.

**Results:**

Baseline ejection fractions were 69.9 ± 5.3% and 76.4 ± 5.4%, and dropped to final values of 40.1 ± 5.8% and 39.4 ± 5.4% in the control and empagliflozin groups, respectively (P < 0.001 *vs.* baseline, P > 0.05 between groups). Collagen deposition, measured as collagen volume fraction, was higher in both the scar and the remote cardiac areas of the control group 79.1 ± 6.2% and 4.6 ± 2.5% for control, and 53.8 ± 5.4% and 2.5 ± 1.3% for empagliflozin group, respectively (P < 0.05 for each). Remote cardiac muscle collagen, measured by hydroxyproline, was 4.1 ± 0.4 μg/μl and 3.6 ± 0.2 μg/μl (P = 0.07). TGF-β1 and Smad3 expression decreased by empagliflozin—18.73 ± 16.32%, 9.16 ± 5.69% and 16.32 ± 5.4%, 7.00 ± 5.28% in the control and empagliflozin groups, respectively (P < 0.05).

**Conclusion/interpretation:**

Empagliflozin administered early after myocardial infarction reduce myocardial fibrosis and inhibit the TGF-β1/Smad3 fibrotic pathway, probably prior to exerting any hemodynamic or physiological effect.

## Introduction

Coronary artery occlusion results in myocardial infarction (MI), myocardial necrosis and subsequent fibrosis, often resulting in decreased cardiac function and heart failure. In the Danish National Patients registry, which include 78,814 patients with MI, 14% had in-hospital heart failure after the index event and additional 5% within the following months, post-MI [1]. The main post-MI pathway leading to heart failure involves extensive negative left ventricular (LV) remodeling, caused by cardiomyocytes’ death and their replacement with a non-functioning fibrotic tissue. Fibrosis is an overgrowth, hardening and scarring of various tissues, and is attributed to excess deposition of extracellular matrix components, mainly collagen [2–5]. Specifically, it is known that fibroblasts, post MI, are transformed into myofibroblast by the macrophages-released transforming growth factor-β1 (TGF-β1) [6], and involved in the fibrotic process. They express high levels of procollagens that enhance collagen deposition in the newly developed scar [7]. Deposition of extracellular proteins, the cornerstone of fibrosis, is a combined and multistep process, involving, pro-inflammatory cytokines such as TGF-β1 and its down-stream effector Smad3 [8, 9].

The new antidiabetic drugs, the sodium-glucose cotransporter 2 inhibitors (SGLT2i), such as empagliflozin, inhibit glucose reabsorption at the nephron's proximal tubule, thus inducing glucosuria and reduce blood glucose level [10]. Recently, empagliflozin has been reported to decrease the risk of severe adverse cardiovascular events, cardiovascular mortality and heart failure admissions [11–13]. Kang et al*.* [14] showed that empagliflozin affects cardiomyocytes to attenuate TGF-β1 expression, which results in cellular protection. Currently, although others studies exhibited empagliflozin’s long term cardiac efficacy in vivo, little is known about the mechanism by which empagliflozin exerts its effect in non-diabetes after MI [15–17].

We hypothesized that early application of empagliflozin post-MI in non-diabetic rats will attenuate collagen deposition and fibrosis by reducing TGF-β1 level and its associated protein Smad3, and eventually result in improved ventricular remodeling.

## Material and methods

### Animals

All animal experiments were conducted according to the institutional animal ethical committee guidelines, which conform to the *Guide for the Care and Use of Laboratory Animals* published by the US National Institutes of Health (Eighth edition 2011) (Ethics 47–07-2019). We used 350–400 gr. (11–12 weeks old) male Sprague–Dawley rats (Envigo Ltd, Jerusalem, Israel), which were maintained at a constant temperature and relative humidity under a regular light–dark schedule (12 h:12 h), fed with normal rodent diet and with tap water ad libitum.

### Study design

All animals underwent baseline cardiac function evaluation by echocardiography, blood pressure (BP) and kidney function. Subsequently, the animals underwent MI procedure. Then, we divided the animals into 2 experimental groups. The first group of untreated rats served as control; the second group received empagliflozin 30 mg/kg/day. Therapy was administered for four consecutive weeks.

We monitored BP once a week and cardiac function was re-evaluated every 2 weeks (week 2 and 4), and kidney function was re-evaluated at the end of the experiment (week 4). Right before sacrifice, we measured cardiac hemodynamics directly by means of the Millar pressure–volume (P–V) system. Finally, after euthanasia, the heart was excised and preserved for additional analyses.

### Echocardiography

We performed echocardiographic scans as previously described [18]. Briefly, under light sedation with 29 mg/kg ketamine and 4.3 mg/kg xylazine, the rats were placed in a left decubitus position and scanned via a commercial echo-scanner (Vivid *i*). Echocardiography at frequency of 9 MHz, depth 2.5 cm and frame rate 315 frames/sec, was used to scan two parasternal short axis sections at the apical (AP) and papillary muscle (PM) levels. Two-dimensional (2D) echocardiography scans took place at baseline before MI, 2 weeks after surgery and at the end of the experiment (4 weeks post-MI). The echocardiographic analysis included diastolic and systolic structural parameters. For ventricular function assessment, we calculated three parameters: (1) Fractional shortening (FS) which takes into consideration a 2D cross-section of the heart. Specifically, in the M-mode cines, the left ventricle (LV) intra-ventricular diameters were measured at systole and diastole (LVIDs and LVIDd, respectively), and FS was calculated as follows: FS = (LVIDd-LVIDs)/LVIDd; (2) Fractional area change (FAC) was calculated as the change in LV area during cardiac cycle. Particularly, LV end diastolic area (LVEDA) and LV end systolic area (LVESA) are measured in 2D cines and FAC were calculated as follows: FAC = (LVEDA-LVESA)/ LVEDA; (3) EF was determined by the Vivid *i* LV function software. The echocardiographic analysis was performed for each level (AP and PM) separately, since the extent of cardiac damage is level specific.

### BP measurements

We monitored BP in conscious rats by a validated tail-cuff plethysmography method using CODA non-invasive BP system (Kent Scientific Corporation, Torrington CT, USA).

### Urine collection

Each rat was placed for 24 h in a metabolic chamber (Techniplast S.p.A., Buguggiate, Italy) in which urine was collected for the determination of its volume, and glucose concentration. Subsequentially, blood sample was withdrawn from the tail vein to asses blood glucose and creatinine levels.

### MI induction

Rats underwent MI procedure, as previously described by Bhindi [19]. Briefly, we intubated the rats under deep anesthesia with a mixture of 87 mg/kg ketamine and 13 mg/kg xylazine and ventilated them at a rate of 80–90 breaths per minute, and 1 to 2 ml/100 gr tidal volume. Using intercostal space left thoracotomy, the chest was opened, and the pericardial sac was dissected. A stich was placed through myocardium at a slightly greater depth than the perceived level of the left anterior descending (LAD) artery. Next, we tightened the suture to ensure complete LAD occlusion, the chest was closed, the skin stitched, and the rat was placed in its cage for recovery.

### Empagliflozin treatment

Empagliflozin powder was generously provided by Boehringer-Ingelheim GmbH, Germany. Drug dosage of 30 mg/kg/day was adopted from Zhou and Wu, as they showed that this rather high dosage affected cardiac physiology as well as cellular biochemistry [20]. Accordingly, rats of group #2 were weighted weekly to adjust their dosage. First drug bolus was given immediately (~ 10–15 min) after MI by subcutaneous injection of the drug dissolved in 0.5 ml DDW. Then, for consecutive 4 weeks, drug was given by 0.5 ml gavage of empagliflozin dissolved in drinking water.

### Direct measurements of cardiac hemodynamics

Direct cardiac hemodynamic parameters measurements were obtained by the Millar P–V system (MPVS-300, Millar Instruments, Houston, TX, USA). The Millar P–V System simultaneously and continuously measures LV pressure and volume from the intact beating heart, producing characteristic P–V loops readings. From the P–V loops the following cardiovascular parameters were derived, systolic and diastolic BPs, heart rate (HR), stroke volume (SV), cardiac output (CO), EF, stroke work (SW), dP/dt_max_ and dP/dt_min_. Briefly, rats were anesthetized with a combination of 87 mg/kg ketamine and 13 mg/kg xylazine and placed on controlled heating pads. Next, the rats were tracheotomized and intubated (0.5 cm long 50 PE tube) to facilitate breathing. The right carotid was exposed and ligated distally, the artery was clamped and incised, a 2-Fr Mikro-Tip® catheter (SPR-838, Millar Instruments, Houston, TX, USA) was advanced through the artery into the LV under pressure control; a ligature was then tightened around the catheter to avoid blood leakage and loss [21]. After stabilization for 5 min, signals were continuously sampled at a sampling rate of 1000 samples/sec by the MPVS-300 system, recorded for 15–20 min, and displayed on a personal computer by the PowerLab System and Chart5 software (AD Instruments, Colorado Springs, CO, USA). At the end of each experiment, 4–6 boluses of 100 μL of hypertonic (30%) saline were injected intravenously, and from the averaged shift of P–V relations, parallel conductance volume (Vp) was calculated by the software and used for the correction of the cardiac mass volume. Thereafter, the catheter was withdrawn, and the animal was sacrificed by overdose anesthetic.

### Organ harvesting‬

After sacrifice, the heart was excised, and dissected transversally at the PM level and its upper section, i.e., AP was fixed in 4% buffered formaldehyde and then embedded in paraffin for histology.

### Histology

Five μm sections were stained with hematoxylin and eosin, photographed and analyzed for structural measurements: Cross-sectional area, LV cross-sectional area, LV cavity area, and septal and anterior wall widths using the ImageJ freeware (NIH, Bethesda, MD, USA).

Picro-Sirius red was used to distinct collagen from healthy muscle in the histological sections thus evaluate collagen deposition. Briefly, after de-paraffinization and re-hydration, we rinsed the slides in distilled water and stained nuclei with Weigert’s hematoxylin solution for 5 min. Then, we placed the slides in Picro-Sirius red stain for 60 min. Next, we rinsed in 2 changes of 0.5% acetic acid water followed by dehydration quickly through 3 changes of absolute alcohol and xylene. Finally, slides covered with coverslip using a permanent mounting medium. Collagen area was measured in scar and remote regions under microscope (Nikon Eclipse Ci-L, Nikon Corporation, Tokyo, Japan) using the NIS Elements software (NIS Elements 4.0, Nikon Corporation, Tokyo, Japan).

We assessed the mean collagen area of 6 fields from each slide, for each photograph, the red color (collagen), the total tissue area and total picture size were measured. Subsequently, we calculated the percentage of the collagen volume fraction (CVF) according to the formula:

$$\% {\text{CVF}} = \frac{{collagen~area}}{{\left( {total~picture~size - ~non~tissue~area} \right)}}{\text{*}}100$$

### Immunohistochemical staining

Sections of myocardial tissue were dewaxed and rehydrated in a graded alcohol series. Antigen retrieval was achieved by heating the specimens for 10 min at 94 °C in citric acid buffer (0.01 mol/l; pH 6.0). Sections were then incubated with 3% hydrogen peroxide at room temperature for 20 min to inactivate endogenous peroxidase activity. After rinsing with PBS buffer (pH 7.2), primary antibodies against TGFβ1 (1:200; cat. no. Ab92486; abcam, Cambridge, MA, USA) or Smad3 (1:200; cat. no. Ab40854; abcam, Cambridge, MA, USA), were added and incubated at room temperature for 1 h. Following further rinsing, anti-rabbit horseradish peroxidase-conjugated secondary antibody (1:1,000; cat. no. D13-6; GBI Labs, Bothell, WA, USA) was added and incubated at room temperature for 15 min. Samples were subsequently incubated with DAB solution at room temperature for 6 min until color development. A total of 5 different fields of view were randomly selected under a light microscope with magnification of × 400. The average absorbance of TGF-β1 was analyzed using the NIS-Elements Software BR analysis system version 4.10.00 (NIS Elements 4.0, Nikon Corporation, Tokyo, Japan).

### Collagen concentration measurement

Collagen concentration was estimated in remote MI LV muscle samples by measuring the hydroxyproline (HOP) concentration [22]. Briefly, frozen cardiac samples were minced and then hydrolyzed at 120℃ for 20 min in 1N HCl, 450 μl of Chloramine-T was added to the hydrolysate, oxidation continued for 25 min in RT. We added 500 μl of Ehrlich’s aldehyde reagent to the samples and incubated at 65℃ for 20 min. Optical density was measured at 550 nm. All samples were measured in duplicates.

### Statistical analysis

Data are presented as mean ± SD. Comparisons between groups were performed by 2-way analysis of variance (ANOVA) with repeated measures, in which the treatment and the time point were the independent variables. Whenever the ANOVA was significant, a multiple comparison was performed using the Holm-Sidak as *post-hoc* test. Single measured data i.e., final measurements, P–V loop derived parameters, and histological analysis were compared between groups using student's *t*-test. P value of < 0.05 was considered significant.

## Results

The initial average body weights were 374 ± 35 and 403 ± 37 gr., in the control and empagliflozin group, respectively. While the control group gained 25 ± 18 gr throughout the experiment, reaching 399 ± 20 gr, the empagliflozin group lost 21 ± 14 gr at final measurement reaching 382 ± 21 gr (P < 0.01 vs. control, Table 1). Still, we found no differences in the heart weight, or the heart to body weight ratio between the groups (Table 1).

**Table 1:** Body and organ weight

Group	Body weight [gr.]	ΔBody weight [gr.]	Heart weight [gr.]	HW/BW [mg/gr.]
Control (n = 6)	399 ± 46	24.8 ± 17.9	1.34 ± 0.15	3.33 ± 0.45
Empagliflozin (n = 8)	382 ± 21	− 11.0 ± 24.9 **	1.24 ± 0.14	3.27 ± 0.52

Values are presented as mean ± SD, ** P < 0.01 *vs.* Control, by student's *t-*test

### Echocardiography

Tables 2, 3 and 4 summarize the echocardiographic parameters obtained at baseline, 2 weeks, and 4 weeks (at animals' sacrifice) post-MI, respectively. At baseline, cardiac structure and function were typical of healthy young rats, i.e., somewhat smaller areas of the AP level vs. the PM level attributing for the conical shape of the LV (Table 1). The AP level is the cardiac territory mostly affected by MI, and therefore herein we describe relevant changes of echocardiography at both levels, distinctively.

**Table 2:** Physical and 2DE structural and functional parameters at baseline

	Level	Parameter	Group	
			Control	Empagliflozin
		Body weight [gr]	413 ± 35	395 ± 37
Apical level	Systolic	LVESA [mm^2^]	16.4 ± 5.9	15.8 ± 5.3
		LVIDs [mm]	4.2 ± 0.7	4.6 ± 0.7
	Diastolic	LVEDA [mm^2^]	43.3 ± 4.9	39.2 ± 5.4
		LVIDd [mm]	7.6 ± 0.6	7.0 ± 0.6
	Function	FS [%]	39.7 ± 3.9	35.6 ± 3.8
		FAC [%]	63.1 ± 3.7	59.0 ± 3.7
		EF [%]	74.5 ± 6.4	70.4 ± 6.2
Papillary muscles level	Systolic	LVESA [mm^2^]	20.5 ± 4.3	26.2 ± 4.3
		LVIDs [mm]	4.3 ± 0.6	4.9 ± 0.7
	Diastolic	LVEDA [mm^2^]	51.2 ± 4.7	47.1 ± 4.6
		LVIDd [mm]	7.5 ± 0.6	7.3 ± 0.5
	Function	FS [%]	36.2 ± 3.1	39.7 ± 3.2
		FAC [%]	52.3 ± 3.6	56.2 ± 4.0
		EF [%]	69.9 ± 5.3	76.4 ± 5.4

LVESA, LVEDA—LV end systolic and end diastolic area, respectively. LVIDs, LVIDd—LV end systolic and end diastolic internal diameter, respectively. *FS﻿* fractional shortening, *FAC* fractional area change, *EF* ejection fraction

**Table 3.** 2DE structural and functional parameters at 2 weeks

	Level	Parameter	Group	
			Control	Empagliflozin
Apical level	Systolic	LVESA [mm^2^]	47.5 ± 4.3^***^	54.4 ± 5.1^***^
		LVIDs [mm]	7.1 ± 0.4^***^	7.9 ± 0.5^***^
	Diastolic	LVEDA [mm^2^]	69.3 ± 4.4^***^	74.3 ± 5.3^***^
		LVIDd [mm]	8.8 ± 0.4^***^	9.3 ± 0.4^***^
	Function	FS [%]	19.1 ± 3.3^**^	15.1 ± 4.0^**^
		FAC [%]	33.6 ± 3.0^***^	27.2 ± 3.5^***^
		EF [%]	42.9 ± 5.4^**^	34.9 ± 6.4^**^
Papillary muscles level	Systolic	LVESA [mm^2^]	47.1 ± 3.4^***^	52.4 ± 4.0^***^
		LVIDs [mm]	7.8 ± 0.4^***^	7.5 ± 0.5^***^
	Diastolic	LVEDA [mm^2^]	78.4 ± 3.8^***^	80.2 ± 4.5^***^
		LVIDd [mm]	9.5 ± 0.2^***^	9.3 ± 0.3^*** #^
	Function	FS [%]	17.8 ± 2.7^***^	20.1 ± 3.2^**^
		FAC [%]	39.3 ± 2.9^***^	35.7 ± 3.4^**^
		EF [%]	40.2 ± 4.6^***^	45.4 ± 5.4^**^

Abbs. as in Table 2. ** P < 0.01 *vs.* baseline and *** P < 0.001 *vs.* baseline, ^#^ P < 0.05 *vs.* damage group

**Table 4:** Physical and 2DE structural and functional parameters at 4 weeks (final)

	Level	Parameter	Group	
			Control	Empagliflozin
		Body weight [gr]	398 ± 46	382 ± 21
Apical level	Systolic	LVESA [mm^2^]	52.4 ± 5.4^***^	53.5 ± 5.1^***^
		LVIDs [mm]	7.9 ± 0.5^***^	7.8 ± 0.5^***^
	Diastolic	LVEDA [mm^2^]	69.3 ± 5.6^**^	77.1 ± 5.3^***^
		LVIDd [mm]	9.5 ± 0.5^**^	9.0 ± 0.4^*^
	Function	FS [%]	17.6 ± 4.2^**^	13.7 ± 4.0^**^
		FAC [%]	27.6 ± 3.8^***^	31.0 ± 3.5^***^
		EF [%]	37.3 ± 6.8^**^	32.3 ± 6.4^**^
Papillary muscles level	Systolic	LVESA [mm^2^]	60.6 ± 4.4^***^	57.5 ± 4.0^***^
		LVIDs [mm]	8.1 ± 0.5^***^	8.2 ± 0.5^***^
	Diastolic	LVEDA [mm^2^]	89.2 ± 4.8^**^	85.3 ± 4.5^***^
		LVIDd [mm]	9.8 ± 0.3^***^	9.8 ± 0.3^***^
	Function	FS [%]	18.6 ± 3.4^***^	17.6 ± 3.2^***^
		FAC [%]	34.1 ± 3.7^**^	33.5 ± 3.4^**^
		EF [%]	40.1 ± 5.8^***^	39.4 ± 5.4^**^

Abbs. as in Table 2. * P < 0.05 *vs.* baseline, ** P < 0.01 *vs.* baseline and *** P < 0.001 *vs.* baseline

Two weeks post-MI, at both cardiac levels (AP and PM) and in both experimental groups (control and empagliflozin treated), LV size increased (P < 0.001 vs. baseline. Table 3), and the functional parameters, i.e., FS, FAC and EF decreased (P < 0.001 *vs.* baseline, Table 3). Collectively, these results indicate that we created an extensive MI.

Four weeks post-MI, at both cardiac levels (AP and PM) and in both experimental groups (control and empagliflozin treated), LV structure remained enlarged in comparison to the basal measurement; however, it is comparable to the two weeks measurement, i.e., no additional significant enlargement or functional deterioration was found (Table 4).

### BP and final hemodynamics

**Fig. 1:**
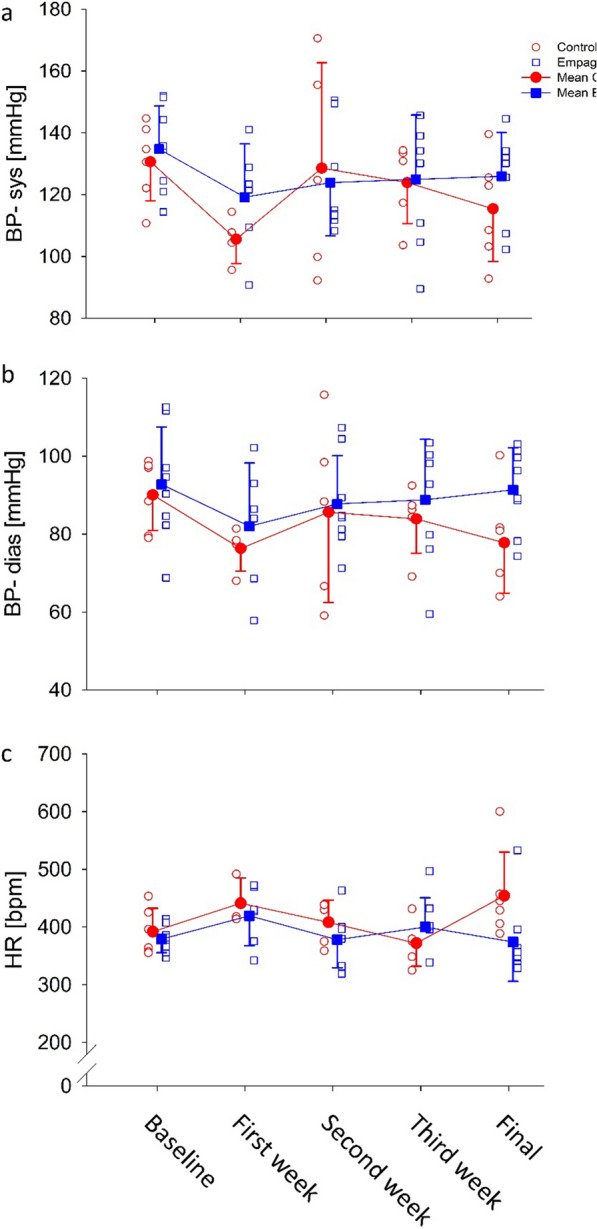
Effect of empagliflozin on BP and HR, **a** BP-systolic; **b** BP-diastolic; **c** HR, red – control group, blue – empagliflozin group. Data presented as individual measurements (empty symbols) and mean ± SD (filled symbols, n=6-8, for each time point) and analyzed using 2-way ANOVA with repeated measures.

Weekly systolic and diastolic BPs and HR are presented in (Fig. 1a–c), respectively, all parameters were comparable between groups throughout the 4 weeks experiment duration (Table 4). Final systolic and diastolic BPs, as well as the parameters derived from the P–V loops recorded by the direct Millar system, are presented in Table 5. All parameters were indistinguishable different between groups, showing no effect of the treatment on cardiac function or BP.

**Table 5:** Hemodynamics and cardiac physiology parameters

Parameter	Group	
	Control	Empagliflozin
Systolic BP [mmHg]	115 ± 17	126 ± 14
Diastolic BP [mmHg]	78 ± 13	91 ± 11
HR [bpm]	454 ± 76	374 ± 68
SV [µl]	98 ± 12	112 ± 23
CO [m/min]	19.7 ± 3.1	22.3 ± 3.8
EF [%]	23.2 ± 2.7	25.8 ± 6.2
SW [mmHg*µl]	6680 ± 2208	7175 ± 2822
dp/dt _max_ [mmHg/sec]	5252 ± 2011	5453 ± 441
dp/dt _min_ [mmHg/sec]	− 3324 ± 1547	− 3417 ± 771

*BP* blood pressure, *HR* Heart rate, *SV* stroke volume, *CO* cardiac output, *EF* ejection fraction, *SW* stroke work, *dp/dt max and min* rate of pressure change at systole and diastole, respectively

### Urine glucose and creatinine

**Fig. 2:**
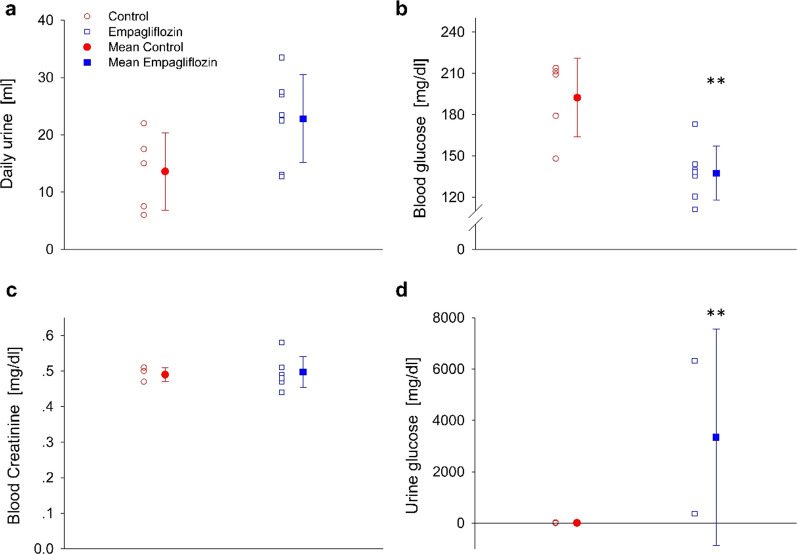
Effect of empagliflozin on urine and blood parameters. **a** Daily urine output, **b** Blood glucose level, **c** Blood creatinine concentration, **d** Urine glucose concentration. Data presented as individual measurements (empty symbols) and mean ± SD (filled symbols, n=6 and 8 to the control and empagliflozin, respectively), control (red), empagliflozin (blue). ** P<0.01 vs. control, *** P<0.001 vs. control, by student's t-test.

Urine volume, glucose concentration and blood creatinine data, as indicators of empagliflozin effect 4 weeks post-MI, are presented in (Fig. 2). As expected, empagliflozin treatment increased daily urine output, (P = 0.06), decreased blood glucose and increased urine glucose output, without affecting creatinine clearance (Fig. 2).

### Histology

**Fig. 3:**
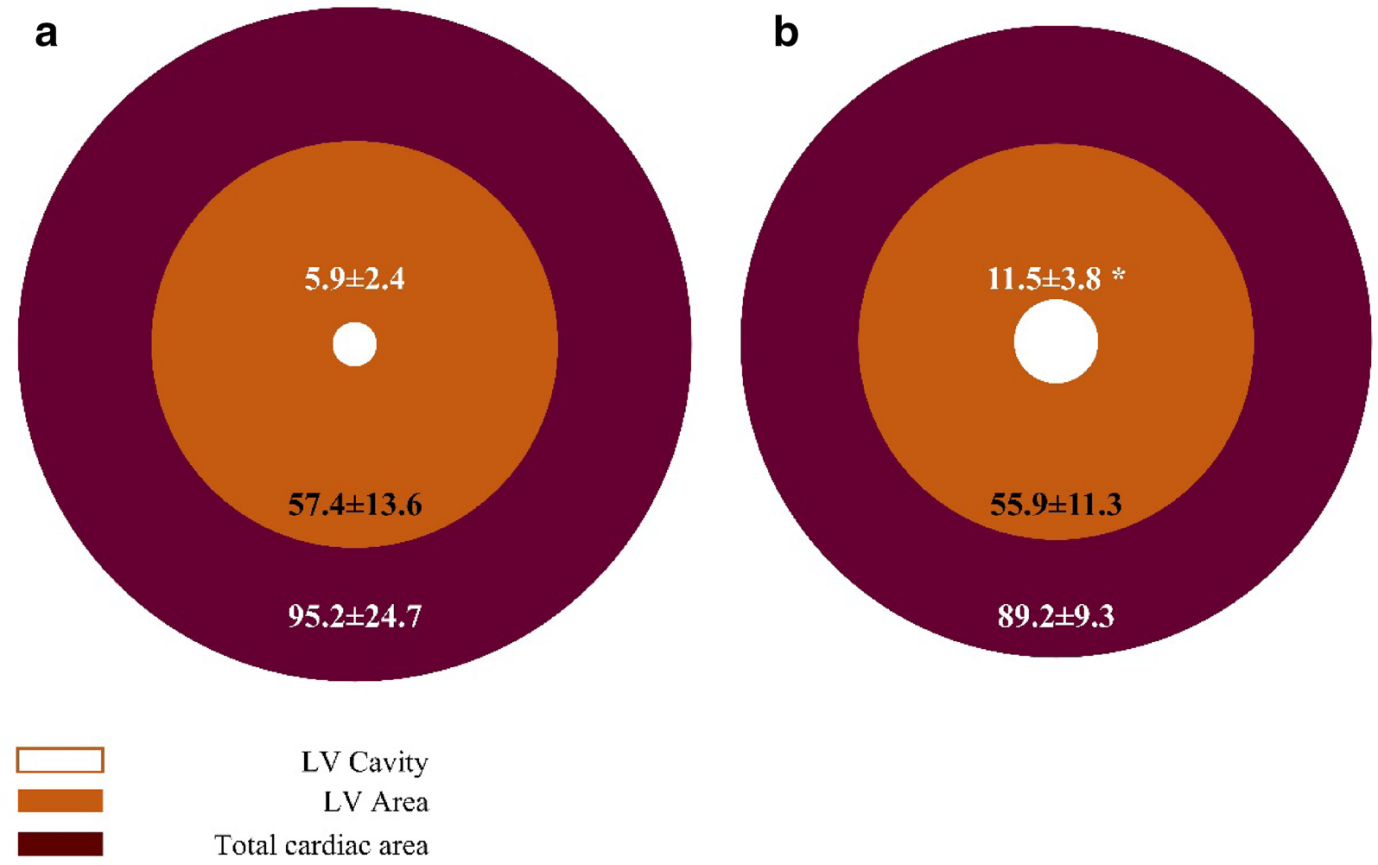
Empagliflozin increases LV cavity without affecting cardiac muscle mass. **a** Control cardiac muscle, **b** Empagliflozin treated cardiac muscle. External circle (deep red) represents total heart cross sectional area, middle circle (orange) represents LV cross sectional area, inner circle (white) – LV cavity cross sectional area. N were 6 and 8 to the control and empagliflozin, respectively * P<0.05 vs. control by student's t-test. Numerical data presents mean±SD of the cross sectional areas.

Gross histological analysis at the PM level reveals a comparable size of the cardiac muscle in both groups (Fig. 3). Specifically, the average total cardiac muscle cross sectional area was 95.2 ± 24.7 mm^2^ and 89.2 ± 9.3 mm^2^, of which LV occupied 57.4 ± 13.6 mm^2^ and 55.9 ± 11.3 mm^2^ in the control and empagliflozin group, respectively (P > 0.05). The LV cavity cross sectional area, on the other hand, was 5.9 ± 2.4 mm^2^ and 11.5 ± 3.8 mm^2^ in the control and the empagliflozin group, respectively (P < 0.05). Thus, while most cardiac structural parameters were comparable, the LV cavity increased in the empagliflozin group.

**Fig. 4:**
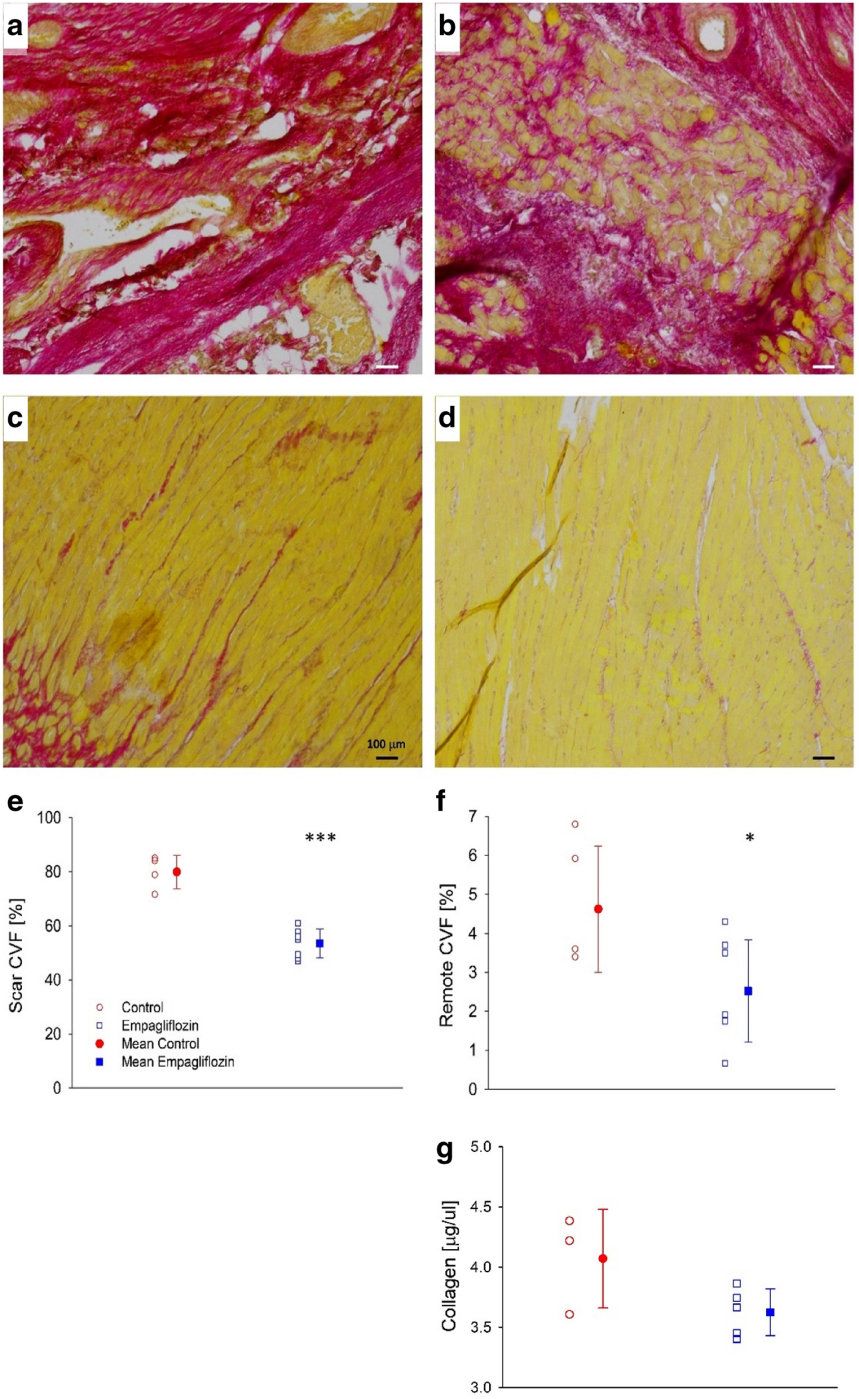
Empagliflozin attenuates post MI collagen deposition. **a** Representative photograph of scar area taken from control rat's heart. **b** Representative photograph of scar area taken from empagliflozin treated rat's heart. **c** Representative photograph of remote area taken from control rat's heart. **d** Representative photograph of remote area taken from empagliflozin treated rat's heart. **e** Collagen volume fraction (CVF) at the scar area. **f** CVF at the remote area. Individual measurements (empty symbols) and average (filled symbols, n = 6 and 8 to the control and empagliflozin, respectively), control (red) and Figure Click here to access/download;Figure;Figures..docx empagliflozin (blue). * P < 0.05 vs. control, *** P < 0.001 vs. control, by student's t-test. Photographs magnification x 40.

Figure 4 depicts sections of cardiac muscles stained for collagen content using picro-sirius red. In these representative Figs., collagen occupies large areas of the scar in the control group, seen as red background with yellow patches of non-collagenous tissue (Fig. 4a). Scars are less collagenous in the treated group, seen as less red color in the empagliflozin representative sections, and more yellow, non-collagenous, patches of tissue (Fig. 4b). In remote areas, collagen is sporadic in both groups, with more collagen in the control group and rare red stain in the empagliflozin group (Fig. 4c, d). CVF quantification in the two regions of the heart, i.e. the scar itself and at remote areas, is presented in (Fig. 4e, f), respectively. The results indicate that CVF decreased by ~ 30% in the scar (P < 0.001), and ~ 45% in the remote area (P < 0.05).

### Hydroxyproline analysis:

Summarizes the HOP concentrations measured at sections of the cardiac muscle. HOP concentrations were 4.1 ± 0.4 µg/µl and 3.6 ± 0.2 µg/µl in the control and empagliflozin treated group, respectively (P = 0.07). These results indicate comparable collagen concentrations in the cardiac muscles of the different experimental groups.

### TGF-β1 and Smad3 expression.

**Fig. 5:**
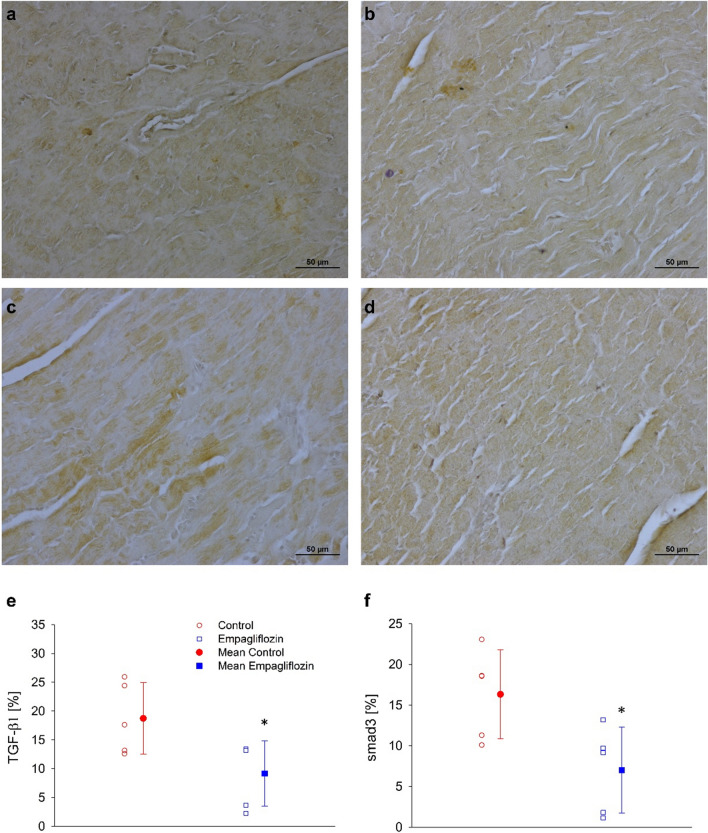
TGF-β1 and Smad3 immuno-histological staining. **a** Representative photograph of immunoprecipitation of anti-TGF-β1 antibody in control cardiac muscle specimen. **b** Representative photograph of immunoprecipitation of anti-TGF- β1 antibody in empagliflozin treated rat's cardiac muscle specimen. **c** Representative photograph of immunoprecipitation of anti-Smad3 antibody in control cardiac muscle specimen. **d** Representative photograph of immunoprecipitation of anti-Smad3 antibody in empagliflozin treated rat's cardiac muscle specimen. **e** Expression of TGF-β1. f. Expression of Smad3. Individual measurements (empty symbols) and average (filled symbols, n=6 and 8 to the control and empagliflozin, respectively), control (red) and empagliflozin (blue). * P < 0.05 vs. control, by student's t-test. Photographs magnification x 200.

As TGF-β1 and Smad3 are key proteins in the fibrosis pathway, we measured their expressions in remote areas of the cardiac muscle by immunohistochemical staining. The representative specimens in (Fig. 5) show reduced expression of both TGF-β1 and Smad3 following empagliflozin treatment (Fig. 5b, d, respectively). Quantification of 5 specimen of each group show that empagliflozin reduced TGF-β1 expression by ~ 49% (P < 0.05*,* Fig. 5e), and Smad3 expression by ~ 47% (P < 0.05, Fig. 5f).

## Discussion

We studied the effect of empagliflozin, a SGLT2i, on myocardial fibrosis and ventricular remodeling early after MI in non-diabetic rats. Rats underwent physiological assessments of cardiac and renal functions, MI induction procedure and 28 consecutive days of empagliflozin (30 mg/kg/day) or saline treatment. Finally, after sacrifice, histological and chemical markers of fibrosis were evaluated. Our results demonstrate that empagliflozin treatment reduced cardiac fibrosis, attenuated collagen deposition (CVF) and decreased TGF-β1 and Smad3 expression; however, it did not cause structural or functional changes post-MI.

Initially, the cardiac beneficial effect of empagliflozin was attributed to its renal effects (polyuria and glucosuria) and subsequent hemodynamic effects, i.e. reduced intravascular blood volume and subsequent cardiac volume overload [15]. These effects may ultimately lead to lowered stroke work, cardiac stretch and attenuated remodeling [15]. Kang et al. have shown some of the direct effects of empagliflozin on cardiomyocytes, and others exhibited even its long term (10–12 weeks) cardiac efficacy in vivo, yet little is known about the mechanism by which empagliflozin exerts its effect in diabetes and non-diabetes after MI [14, 16, 17]. It was suggested that empagliflozin affected cellular hypertrophy by attenuated ANP mRNA expression in a hypertension-induced heart failure model [15]. Additionally, empagliflozin efficacy was related to its local anti-inflammatory activity [23]. Moreover, it was found that empagliflozin inhibits the sodium-hydrogen exchanger-1 (NHE-1) in the heart [12, 13].

These mechanisms of action may result in a reduction in cardiac injury, reduction of hypertrophy and fibrosis, attenuation of ventricular remodeling and prevention of systolic dysfunction [12]. However, we did not find this association in our cardiac or hemodynamic data. Thus, an alternative hypothesis regarding empagliflozin mechanism of action need to be considered. To decipher the mechanism of action of empagliflozin, we evaluated its ability to attenuate fibrosis, and the expression of key pro-fibrotic proteins, TGF-β1 and Smad3. Indeed, we show that empagliflozin has an anti-fibrotic effect, as measured by CVF reduction at both the scar as well as remote regions of the heart.

Recent studies have reported that empagliflozin treatment also improves diastolic function in a diabetic and non-diabetic heart failure model with preserved ejection fraction (EF), as well as after MI [15, 24, 25]. These studies attributed empagliflozin efficacy to its ability to reduce cardiac volume overload, and improve mitochondrial physiology and function, respectively [15, 24]. Interestingly, Connelly et al. in a rat model of MI, reached almost similar results to ours, i.e. reduction of fibrosis, without improvement of cardiac function [21]. They related empagliflozin efficacy not just to its ability to reduce cardiac preload, thus hinting to other mechanisms of action [21]. These could be anti-fibrotic, anti-inflammatory or other mechanisms. Lee et al. in their hypertension induced heart failure model, associated empagliflozins’ efficacy to its anti-inflammatory effects [23]. It is worth mentioning, that in Connelly’s et al. study, empagliflozin treatment was given late (a week post-MI), and that the Lee et al. study, involved a rather mild remodeling model, i.e. only hypertension. We found, anti-fibrotic effects of empagliflozin, given early post-MI, without cardiac function improvement. Santos-Gallego et al*.* showed that a long-term (2 months after MI) empagliflozin treatment given to big mammals (swines) improved cardiac function as well as reduced TGF-β1 and Smad3 levels.

In summary, we suggest that CVF reduction may evolve from a direct effect of empagliflozin on the cardiac muscle and the inhibition of the TGF-β1/Smad3 pathway, as Kang et al. showed in vitro. In their study, empagliflozin significantly attenuated TGF-β1-induced fibroblast activation after 72-h exposure, resulting in less matrix remodeling [14]. Yet, the mechanism of action was not thoroughly deciphered. Here, in a small mammal (rat) and one-month post-MI, we show mainly a biochemical, rather than a physiological effect of empagliflozin. Together, the in vitro study by Kang et al*.*, and our in vivo study provide data showing a direct effect of empagliflozin on cardiac fibrosis by attenuation collagen deposition, fibrosis and cardiac remodeling through the TGF-β1/Smad3 pathway. We, therefore, assume that the effect of empagliflozin may be independent of its physiological effect, as it precedes the latter. Yet, one cannot exclude the possibility that other pathways may be involved in the efficacy of SGLT2is. Lee et al. has shown previously that dapagliflozin, for example, had an in vivo efficacy in rats, attenuating cardiac remodeling and preserving its function [26].

### Clinical implication

A summary of the possible pathways involving the SGLT2is efficacy, must include references to the direct physiological aspect of the drugs, i.e. reduction of blood glucose level, increasing urine output, and to possible biochemical pathways such as the STAT3, NHE-1 inhibition and the TGFβ1/Smad3 pathways [27–29]. Clinically, in diabetic patients, six months of empagliflozin treatment did not significantly improve left ventricular (LV) function, structure, adiposity, or diffuse fibrosis [29]. The DAPA-HF trial [30], and EMPEROR-REDUCED trial [31], showed a significant reduction of cardiovascular adverse events in diabetic as well as non-diabetic patients with reduced LV function. Further, it was established that empagliflozin causes reverse remodeling [32, 33] and improves LV systolic function in HFrEF patients [33].

Thus, it is still not clear whether and how SGLT2is affect the cardiovascular system. However, empagliflozin and other SGLT2is exerts a physiological effect at the renal level, i.e., it increases daily urine output and glucose excretion, lowers blood glucose and body weight gain. The former, reduces hyperglycemia, and attenuate its related oxidant production, i.e. exerts antioxidant efficacy. The latter, affects volume overload reducing cardiovascular general and particularly cardiac stresses, thus attenuate their malfunction remodeling.

Other biochemical pathways were established both in vitro and in vivo, among them the STAT3, cardiac and renal NHE-1 inhibition, and the TGFβ1/Smad3 pathways [34] The STAT3 is known for its anti-inflammation efficacy. Lee et al. [25] have shown in vivo, that the SGLT2i, dapagliflozin, preserved cardiac function and attenuate remodeling in non-diabetic rats, attributing these effect to the inhibition of the above-mentioned mechanism. NHE-1 inhibition, is another mechanism attributed to the SGLT2is, specifically it was shown that those agents inhibit the NHE-1 function, which its function is associated with cardiac function deterioration and remodeling in the diseased heart [27, 28].

We tested the in vivo effect of empagliflozin on the TGFβ1/Smad3 pathway, which is involved in the collagen formation, as was shown by Kang et al. [14]. However, our results failed to show physiological efficacy in our MI non-diabetic rats. Finally, those biochemical pathways were not yet established in diabetic or non-diabetic patients, yet it is reasonable to believe that they do affect patients who receive SGLT2i.

## Study limitations

We used significantly higher doses of empagliflozin than those currently used in clinical practice, yet those doses acceptable in the pre-clinical arena. We showed a significant impact on scar formation and TGF-β1/Smad3 expression, without a significant difference in ventricular function or hemodynamics between the groups. Additional echocardiographic parameters such as E/A ratio, E/e' ratio, would have might shed light regarding diastolic function, unfortunately, those could not be derived from the cines of this research. It is possible, however, that longer treatment duration (> 10 weeks) would result in a significant preservation of cardiac structure and function as seen by Santos-Gallego et al. who administered empagliflozin for 2 months. Clinical trials such as EMPEROR-REDUCED, showed that the combined cardiovascular death and hospitalization was reduced significantly only after more than 90 days (~ 21 weeks) of treatment, which correspond to ~ 10 weeks of treatment in rats. Further, we performed this research in small mammals (rats). Their physiology, biochemistry and the drug efficacy may differ from bigger mammals (swine) or humans.

Other parameters of cardiac damage, such as BNP, could have been studied as well; however, we did not sample them in the current setup. Other experimental groups that could have been included in the research are sham group (i.e., rats that underwent MI, without damage induction) and a group treated with empagliflozin to represent the net effect of empagliflozin, yet both such groups were already studied in other setups.

## Conclusions

Our study shows that empagliflozin, given early post-MI, attenuates collagen content and fibrosis by inhibiting the TGF-β1/Smad3 expression. However, we did not find a beneficial effect of empagliflozin on ventricular function. The results of our research should encourage additional studies in this direction involving other doses, treatment duration, and perhaps milder damage models.

## Data Availability

Not applicable.

## Abbreviations

AP: Apical
CO: Cardiac Output
EF: Ejection fraction
FAC: Fractional area change
FS: Fractional shortening
HR: Heart rate
LAD: Left Anterior Descending
LV: Left Ventricle
LVEDA: LV End Diastolic Area
LVESA: LV End Systolic Area
LVIDs: LV Intra-Ventricular Diameters at Systole
LVIDd: LV Intra-Ventricular Diameters at Diastole
MI: Myocardial infarction
NHE-1: Sodium-Hydrogen Exchanger-1
PM: Papillary muscle
P–V: Pressure–volume
SGLT2i: Sodium-Glucose Cotransporter 2 Inhibitors
SV: Stroke volume
SW: Stroke work
TGF-β: Transforming Growth Factor-β
